# Predicting Cognitive Decline in Older Adults Using Baseline Metrics of AD Pathologies, Cerebrovascular Disease, and Neurodegeneration

**DOI:** 10.1212/WNL.0000000000201572

**Published:** 2023-02-21

**Authors:** Lloyd Prosser, Amy Macdougall, Carole H. Sudre, Emily N. Manning, Ian B. Malone, Phoebe Walsh, Olivia Goodkin, Hugh Pemberton, Frederik Barkhof, Geert Jan Biessels, David M. Cash, Josephine Barnes, for the Alzheimer's Disease Neuroimaging Initiative

**Affiliations:** From the Department of Neurodegenerative Disease (L.P., A.M., C.H.S., E.N.M., I.B.M., P.W., H.P., D.M.C., J.B.), Dementia Research Centre, UCL Queen Square Institute of Neurology, London; Medical Statistics (A.M.), London School of Hygiene and Tropical Medicine; School of Biomedical Engineering and Imaging Sciences (C.H.S.), King's College London; Centre for Medical Image Computing (C.H.S., O.G., H.P., F.B.) and Department of Population Sciences and Experimental Medicine (C.H.S.), MRC Unit for Lifelong Health and Ageing at UCL, University College London, United Kingdom; Department of Radiology and Nuclear Medicine (F.B.), VU University Medical Center, Amsterdam Neuroscience; and Department of Neurology and Neurosurgery (G.J.B.), UMC Utrecht Brain Center, University Medical Center Utrecht, the Netherlands.

## Abstract

**Background and Objectives:**

Dementia is a growing socioeconomic challenge that requires early intervention. Identifying biomarkers that reliably predict clinical progression early in the disease process would better aid selection of individuals for future trial participation. Here, we compared the ability of baseline, single time-point biomarkers (CSF amyloid 1–42, CSF ptau-181, white matter hyperintensities (WMH), cerebral microbleeds, whole-brain volume, and hippocampal volume) to predict decline in cognitively normal individuals who later converted to mild cognitive impairment (MCI) (CNtoMCI) and those with MCI who later converted to an Alzheimer disease (AD) diagnosis (MCItoAD).

**Methods:**

Standardized baseline biomarker data from AD Neuroimaging Initiative 2 (ADNI2)/GO and longitudinal diagnostic data (including ADNI3) were used. Cox regression models assessed biomarkers in relation to time to change in clinical diagnosis using all follow-up time points available. Models were fit for biomarkers univariately and together in a multivariable model. Hazard ratios (HRs) were compared to evaluate biomarkers. Analyses were performed separately in CNtoMCI and MCItoAD groups.

**Results:**

For CNtoMCI (n = 189), there was strong evidence that higher WMH volume (individual model: HR 1.79, *p* = 0.002; fully adjusted model: HR 1.98, *p* = 0.003) and lower hippocampal volume (individual: HR 0.54, *p* = 0.001; fully adjusted: HR 0.40, *p* < 0.001) were associated with conversion to MCI individually and independently. For MCItoAD (n = 345), lower hippocampal (individual model: HR 0.45, *p* < 0.001; fully adjusted model: HR 0.55, *p* < 0.001) and whole-brain volume (individual: HR 0.31, *p* < 0.001; fully adjusted: HR 0.48, *p* = 0.02), increased CSF ptau (individual: HR 1.88, *p* < 0.001; fully adjusted: HR 1.61, *p* < 0.001), and lower CSF amyloid (individual: HR 0.37, *p* < 0.001; fully adjusted: HR 0.62, *p* = 0.008) were most strongly associated with conversion to AD individually and independently.

**Discussion:**

Lower hippocampal volume was a consistent predictor of clinical conversion to MCI and AD. CSF and brain volume biomarkers were predictive of conversion to AD from MCI, whereas WMH were predictive of conversion to MCI from cognitively normal. The predictive ability of WMH in the CNtoMCI group may be interpreted as some being on a different pathologic pathway, such as vascular cognitive impairment.

Dementia affects over 50 million people worldwide, making it one of the greatest socioeconomic challenges of our time.^1^ Of dementia cases, around 50%–75% will have a primary diagnosis of Alzheimer disease (AD), with high proportions of cases with mixed pathologies at postmortem.^2^ Early diagnosis and prediction of clinical progression is imperative because earlier intervention, before significant decline in cognition, is likely to lead to more effective treatment. Identifying biomarkers that are predictive of clinical progression in those without initial cognitive impairment and those with mild cognitive impairment (MCI) would better aid selection of individuals for future trial participation. As biomarkers represent different pathologic processes, assessing their individual and independent predicative ability above others would further inform our understanding of complexities of progression in both MCI and AD.

There is a general consensus that biomarkers representing hallmark pathologies in AD, such as extracellular cerebral amyloid deposition and intracellular phosphorylated tau tangle accumulation, precede neurodegeneration biomarkers.^3^ In clinical AD, these biomarkers have been estimated to deviate approximately 10–15 years before the earliest signs of cognitive impairment,^4^ with some reports of amyloid changing before tau. CSF biomarkers of amyloid and tau agree well with postmortem amyloid deposition and tau accumulation, respectively.^5^ CSF amyloid beta (1–42) and phosphorylated tau 181 (ptau) have varied reported abilities in predicting clinical change in healthy controls. In univariate models, both amyloid and ptau have been shown to have significant associations with progression to MCI symptom onset,^6^ but when modeled with whole-brain volumes, only higher ptau levels have been shown to have associations with progression.^7^ Additional reports that binarize CSF amyloid and ptau into positive and negative groups found being amyloid or ptau positive was associated with progression to AD or dementia, with CSF amyloid showing increased group separation over ptau.^8^

A well-established downstream biomarker of neurodegeneration in dementia is brain atrophy.^9^ Previous studies using Cox regression modeling in participants with MCI found that single time-point whole-brain and hippocampal volumes were predictive of future progression to AD.^10,11^ Some findings showed that whole-brain volume, hippocampal volume, and CSF amyloid were separately predictive of conversion from MCI to AD, whereas in multivariate models, only whole-brain and hippocampal volumes were significant.^12^ There is little information regarding the use of these biomarkers in predicting decline from controls to MCI.

Some reports highlight the coexistence of cerebrovascular disease (CVD) in AD.^13,14^ CVD has numerous imaging features associated with differing underlying pathologies. White matter hyperintensities (WMH) of presumed vascular origin and cerebral microbleeds (CMBs) are 2 such imaging features thought to represent different pathologic processes,^15^ both associated with neurodegeneration.^16,17^ Higher WMH burden at baseline is associated with later progression to MCI,^18^ and increases in WMH volumes have been reported to occur before MCI onset.^19^ WMH have been shown to be associated with MCI symptom onset in individuals with a low level of total tau,^20^ but not when modeled with CSF biomarkers (amyloid and ptau). Because of WMH and CMB presence in AD, and mixed findings in univariate and smaller multivariate models, assessment of these markers in predicting progression separately and in models with biomarkers of AD and neurodegeneration is important.

Cox regression models that assess classical biomarkers typically focus on univariate biomarker models,^8,10,11^ with some exploring the associations of AD-related biomarkers in multivariate models.^7,12,20^ Because multiple pathologies are often present in AD, considering the individual and independent association of these biomarkers is useful. This will further help make inferences about competing markers involved in AD pathology and elucidate biomarkers that are consistently associated with clinical progression.

In this study, we assessed single time-point (baseline) biomarkers of pathology and their separate and independent abilities to predict subsequent clinical progression in those without dementia. Here, we use survival analysis to explore whether CSF biomarkers of AD-related pathology (amyloid and tau), neurodegenerative biomarkers (whole-brain and hippocampal volumes), and measures of CVD (WMH and CMB) are useful in predicting clinical progression. In addition to assessing the individual predictive value of each marker, we fitted a multivariable model including all biomarkers to predict progression to MCI from normal cognition (CN) (CNtoMCI) and AD from MCI (MCItoAD). By focusing on single time-point biomarkers, we aim to evaluate clinical utility of cross-sectional (single visit) measures because longitudinal metrics may not be possible to collect for all individuals.

We hypothesize that predictors of conversion from CN to MCI and from MCI to AD will differ, because of the initial clinical stage of each group. In addition, we hypothesize that multiple biomarkers will be better at predicting conversion than individual biomarkers owing to the heterogeneity of pathologies present in groups.

## Methods

### Cohort

Data used in the preparation of this article were obtained from the AD Neuroimaging Initiative (ADNI) database (adni.loni.usc.edu). ADNI was launched in 2003 as a public-private partnership, led by Principal Investigator Michael W. Weiner, MD. The primary goal of ADNI has been to test whether serial MRI, PET, other biomarkers, and clinical and neuropsychological assessment can be combined to measure the progression of MCI and early AD. For up-to-date information, see adni-info.org.

In this study, newly enrolled ADNI2/GO participants who were CN, or who had MCI, at the baseline assessment (which follows the screening visit), were included. These participants were followed through the course of ADNI2/GO, with some continuing to ADNI3. The CN group included those who were labeled as either CN or significant memory concern (SMC); those who were in the MCI group had either late or early MCI according to the screening visit.

All individuals included in the current study were of good general health and between 55-90 years, spoke either English or Spanish fluently, and had a reliable study partner and a Hachinski score of <5. Only participants with preserved activities of daily living and an absence of any other significant neurologic disorder apart from suspected AD were included.

CN individuals were defined by having a Mini-Mental State Examination (MMSE) score between 24 and 30 (inclusive) at baseline and a Clinical Dementia Rating (CDR) score of 0. CN individuals were normally functioning as measured by education-adjusted scores on delayed recall of 1 paragraph from Wechsler Memory Scale Logical Memory II. CN individuals who reported subjective memory concerns were labeled as SMC. Individuals with MCI were required to have an MMSE score between 24 and 30 (inclusive) at baseline, objective memory loss by education-adjusted scores on Wechsler Memory Scale Logical Memory II, a global CDR equal to 0.5, and report subjective memory concerns.

Individuals were given a diagnosis at baseline, month 6, month 12, and then yearly. Changes in diagnosis were recorded at these time points. At follow-up, those with evidence of clinical progression were given a converting diagnosis by a physician at site, whereas those with improvements may have received a reverting diagnosis. For those progressing from MCI to AD, individuals with AD were defined by having an MMSE score between 20 and 26 (inclusive), a CDR of 0.5 or 1.0, subjective memory concern, and NINCDS-ADRDA criteria for probable AD.

To be included in this study, individuals had to have complete measures of CSF amyloid beta 1–42 and phosphorylated tau 181 (ptau) at their baseline visit and suitable MRI scans that produced quality measures of WMH, CMB, whole-brain, hippocampal, and total intracranial volume (TIV) measurements.

### CSF Measurements

Baseline CSF amyloid 1–42 and ptau 181 measurements (untransformed to ADNI1) were obtained from the ADNI biomarker core (University of Pennsylvania) using the microbead-based multiplex immunoassay, the INNO-BIA AlzBio3 RUO test (Fujirebio, Ghent, Belgium), on the Luminex platform (Luminex Corp, Austin, TX) (UPENN_CSF_Biomarker_Data_Master [ADNI1,GO,2], Version: 2016-07-05).

### Presumed Cerebrovascular Measurements

WMH volumes of presumed vascular origin in the supratentorial brain region were calculated using BaMoS (applied to FLAIR and T1-weighted images).^21^ All outputs were visually assessed by experienced raters.^22^

Numbers of probable and definite microbleeds were identified and counted using the Microbleed Anatomical Rating Scale using T2*-weighted imaging.^23^ Both FLAIR and T1-weighted imaging were registered to the T2*-weighted imaging to ensure accurate identification of microbleeds. For both microbleed identification and the checking of WMH, the software package NiftyMIDAS was used (Centre for Medical Image Computing, UCL).^24^

### Brain Volume Measurements

Whole-brain volume, hippocampal volume, and TIV were extracted from T1-weighted scans. Whole-brain volumes were calculated using semiautomated Brain MAPS,^25^ with quality control and manual edits made using MIDAS.^26^ Quality-controlled hippocampal volumes were calculated using STEPS,^27^ and TIVs were calculated from T1-weighted images using the Geodesic information flows label fusion framework.^28^

### Demographics

Diagnostic and demographic data (age, sex, race, education, and follow-up time) were downloaded from the ADNI database (adni.loni.usc.edu/).

## Statistical Analysis

### Data Transformation

We initially log-transformed (log_2_) WMH and then standardized all biomarkers used in Cox regression models to produce z scores. Standardization enabled consistency and comparisons of the individual biomarkers' ability in predicting change in diagnosis. We further created dichotomized biomarkers using a median split for those biomarkers that showed significant prediction of progression to facilitate the result visualization. Because of its bimodal nature, a gaussian mixture model was used to establish the value of CSF amyloid used to dichotomize results (cutpoint 256 pg/mL). This was performed using R^29^ and has been used previously for this variable.^30^

#### Baseline (First Time Point) Variable Analysis

The CNtoMCI and MCItoAD groups were analyzed separately. For continuous measures, we used linear regression to assess whether those who converted differed from those who remained stable. For WMH, log-transformed WMH were used in the regression models. For binary variables, the Fisher exact test was used.

### Modeling the Prediction of Progression

The CNtoMCI and MCItoAD groups were analyzed separately. Cox proportional hazards regression was used, which can allow for the time-to-event outcome (diagnostic change and named survival probability) and data censoring. Individual models were fitted for each biomarker separately as well as a multivariable (fully adjusted) model.

To establish whether hazards were constant over time, a test for proportional hazards assessment was made. In instances of nonproportional hazards, an interaction of log time was introduced for each nonproportional marker.

Likelihood ratio tests were performed to compare the goodness of fit of different models. A Harrell's C-index was used to assess the predictive power of the fully adjusted compared with univariate models. In models with nonproportional hazards, a weighted C-index was used,^31^ which allows for an average hazard ratio (HR) to be found even in the presence of nonproportional hazards.

In addition to individual and fully adjusted models, we created other exploratory models of different group combinations (eTables 1 and 2, links.lww.com/WNL/C477). These exploratory models included a CSF model (amyloid, ptau), a presumed vascular model (WMH, microbleeds), and a neurodegeneration (volume) model (whole brain, hippocampi).

### Adjustments and Covariates

In the demographics, TIV was included as a nuisance covariate in linear regressions assessing differences in WMH, hippocampal volume, and whole-brain volume across groups. For Cox regression models, age was used as a covariate. Cox regression models that included WMH, hippocampal volume, and whole-brain volume also included TIV as a nuisance covariate. Results from unadjusted Cox regression models (apart from the nuisance variable of TIV) and results from models with age, sex, and education covariates are shown in eTables 3–6, links.lww.com/WNL/C477.

### Visualization of Results

To visualize the data, we created forest plots to show the HR and 95% CIs for each marker modeled separately (using outputs from the individual models) and independently (from the fully adjusted model). For significant biomarkers, we produced Kaplan-Meier curves to show conversion probability over time using dichotomized values described earlier.

### Standard Protocol Approvals, Registrations, and Patient Consents

For ADNI, protocol and informed consent forms were approved by the institutional review board at each participating site.

### Data Availability

Anonymized data are available from ADNI (adni.loni.usc.edu/), and included data will be made available by request from any qualified investigator.

## Results

From an initial sample of 1,217, 661 participants were removed because of incomplete data (n = 395, CNtoMCI; n = 266, MCItoAD). An additional 22 participants were removed owing to fluctuating, reverting, or missing longitudinal diagnoses (n = 3, CNtoMCI; n = 19, MCItoAD).

The remaining 534 individuals (CNtoMCI, n = 189; MCItoAD, n = 345) were included in this study (Table). For the CNtoMCI group, we found that the converters were over 4 years older in age at baseline (*p* < 0.001) and with double the volume of WMH (*p* < 0.001) than those who remained stable and cognitively normal. In this group, the converters had lower CSF amyloid (*p* = 0.002), whole-brain volumes (*p* = 0.001), and hippocampal volumes (*p* < 0.001) compared with those who remained stable. In the MCItoAD group, we found converters to be nearly 2 years older (*p* = 0.04) at baseline on average, with higher levels of CSF ptau (*p* < 0.001) and a greater proportion of individuals with microbleeds (*p* = 0.01). Those who converted from a diagnosis of MCI to AD had lower levels of CSF amyloid (*p* < 0.001), whole-brain volumes (*p* < 0.001), and hippocampal volumes (*p* < 0.001) compared with those who remained stable. Full demographics showing values before exclusion of individuals are reported in eTable 7, links.lww.com/WNL/C477.

**Table:**
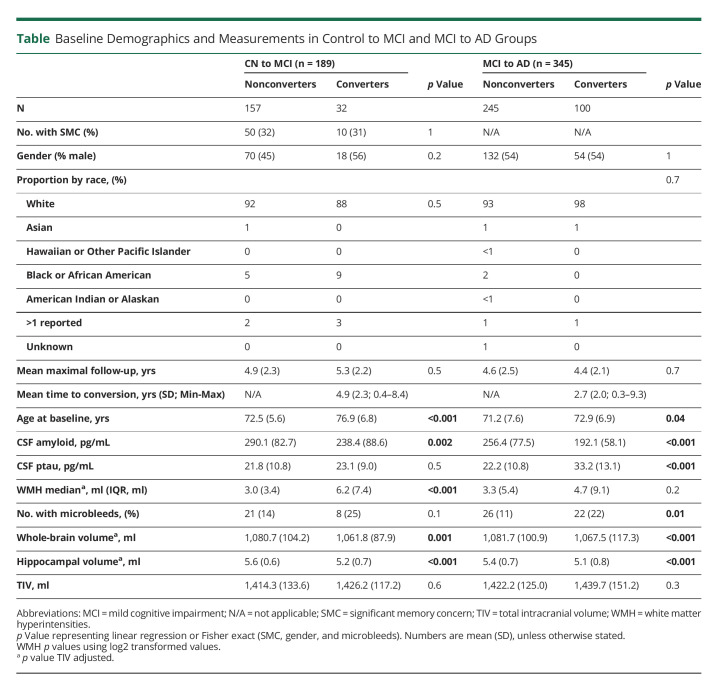
Baseline Demographics and Measurements in Control to MCI and MCI to AD Groups

## CNtoMCI

**Figure 1:**
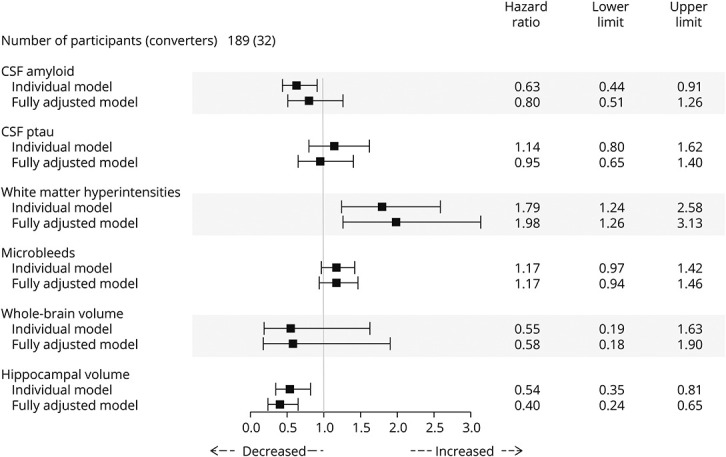
Hazard Ratios in the CNtoMCI Group HR scores (along with upper and lower CI limits) from the Cox regression models separately and in the fully adjusted model (containing all biomarkers), with age adjustment. Individual models, WMH, hippocampal volume, and whole-brain volume, and the fully adjusted model were TIV adjusted. The proportional hazard (PH) assumption was met in all instances (*p* > 0.05, all tests). The individual bars visually represent these HRs along with their uncertainty around the estimate (upper and lower limits). Bars below 1 highlight that decreased levels of the biomarker are associated with progression, whereas bars above 1 highlight that increased levels of the biomarker are associated with progression. Bars crossing 1 are not significantly associated with clinical progression. CN = cognitively normal; HR = hazard ratio; TIV = total intracranial volume; WMH = white matter hyperintensities.

Both separately and in the fully adjusted model, greater WMH burden and lower hippocampal volume were most strongly associated with conversion to MCI from CN (Figure 1). In the fully adjusted model, there was a strong association between both higher WMH (HR 1.98; *p* = 0.003) and lower baseline hippocampal volume (HR 0.40; *p* < 0.001) with conversion. Lower CSF amyloid levels were separately associated with conversion (HR 0.63; *p* < 0.001). There was no evidence that CSF ptau, microbleeds, or whole-brain volume was associated with conversion. Using a likelihood ratio test, there was evidence that the fully adjusted model had a better fit compared with each of the individual models (*p* < 0.004, all tests) and a higher Harrell's C-index of the fully adjusted model (0.77) compared with individual markers.

Additional models of CSF, presumed vascular, and volume are reported in eTable 1, links.lww.com/WNL/C477. CSF amyloid (HR 0.62; *p* = 0.02) was predictive in the CSF model, WMH (HR 1.75; *p* = 0.004) were predictive in the presumed vascular model, and hippocampal volume (HR 0.55; *p* = 0.006) was predictive in the volume model.

**Figure 2:**
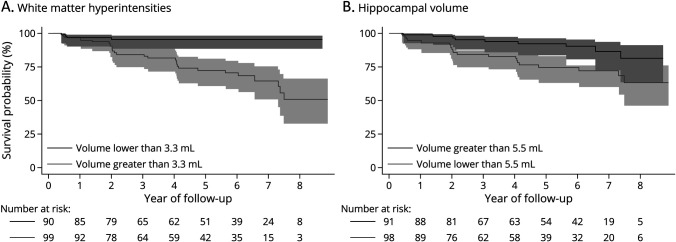
Kaplan-Meier Survival Estimates in the CNtoMCI Group (A and B) Kaplan-Meier curves of the two separately and independently significant predictors in both individual and fully adjusted Cox regression models (WMH and hippocampal volume) for the CNtoMCI group. Continuous variables have been dichotomized at a median point, with shaded regions representing 95% CI. CN = cognitively normal; MCI = mild cognitive impairment; WMH = white matter hyperintensities.

For WMH reported in Figure 2A, the Kaplan-Meier curves suggest a clear distinction between those with WMH values either side of the median value, with those with greater than median values more likely to progress to MCI. For hippocampal volumes reported in Figure 2B, those with lower volumes had a greater conversion rate but with overlap in the 95% CIs of the estimates at every time point.

## MCItoAD

**Figure 3:**
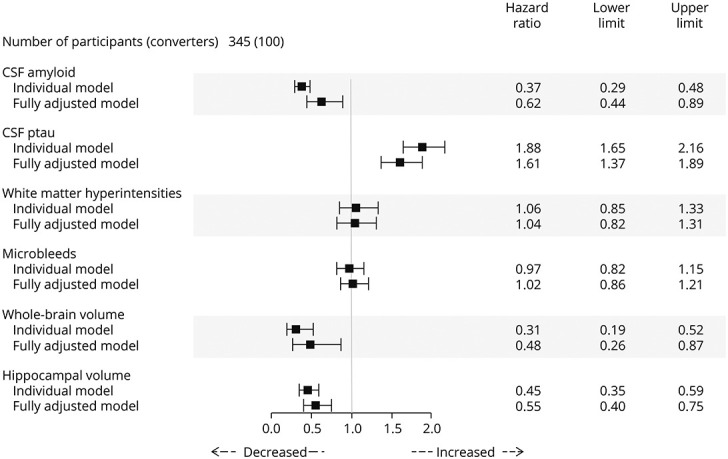
Hazard Ratios in the MCItoAD Group Hazard ratios and their upper and lower limits from both individual and fully adjusted models for the MCItoAD group with age adjustment. Individual models, WMH, hippocampal volume, and whole-brain volume, and the fully adjusted model were TIV adjusted. The proportional hazards assumption was met in all individual models (*p* > 0.05, all tests) excluding hippocampal volume, so a time varying coefficient (TVC) was included to account for this. In the fully adjusted model, this assumption was not met for whole-brain volume or for hippocampal volume (*p* < 0.05, both tests) and was accounted for with a TVC correction. A TVC correction was also applied to age. The individual bars visually represent these HRs along with their uncertainty around the estimate (upper and lower limits). Bars below 1 highlight that decreased levels of the biomarker are associated with progression, whereas bars above 1 highlight that increased levels of the biomarker are associated with progression. Bars crossing 1 are not significantly associated with clinical progression. AD = Alzheimer disease; HR = hazard ratio; MCI = mild cognitive impairment; WMH = white matter hyperintensities.

Lower hippocampal volume, whole-brain volume, CSF amyloid, and higher CSF ptau were most strongly associated with later conversion from MCI to AD both separately and independently (Figure 3). There was evidence of a strong association of conversion with higher CSF ptau (HR 1.61; *p* < 0.001), lower hippocampal volume (HR 0.55; *p* < 0.001), lower CSF amyloid (HR 0.62; *p* = 0.008), and lower whole-brain volume (HR 0.48; *p* = 0.008) in the fully adjusted model. The fully adjusted model had a significantly better fit than all individual models (*p* < 0.001, all tests) and a higher weighted Harrell's C-index of the fully adjusted model (0.81) compared with individual markers.

eTable 2, links.lww.com/WNL/C477, shows predictive abilities of biomarkers in other models (CSF, presumed vascular, and volume). Consistent with the individual and fully adjusted models, lower hippocampal volume and whole-brain volume, higher CSF ptau, and lower CSF amyloid were most strongly associated with later conversion from MCI to AD.

**Figure 4:**
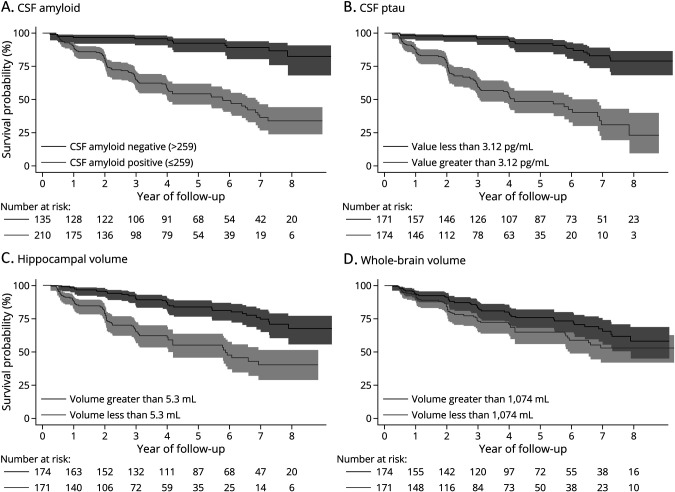
Kaplan-Meier Survival Estimates in the MCItoAD Group (A–D) Kaplan-Meier curves of the three separately and independently significant predictors in both individual and fully adjusted Cox regression models (CSF amyloid, CSF ptau, hippocampal volume, and whole-brain volume), displaying their individual predictive power over time by median split or gaussian mixture model cutpoint (raw CSF amyloid cutpoint of 256 pg/mL). Shaded regions represent 95% CI. AD = Alzheimer disease; MCI = mild cognitive impairment.

The Kaplan-Meier curves (Figure 4A) show a clear distinction between CSF amyloid–positive and CSF amyloid–negative individuals, with amyloid-positive individuals more likely to progress to AD. CSF ptau Kaplan-Meier curves show that those with greater than median values were more likely to convert to AD (Figure 4B). For hippocampal volume, those with volumes below the median value were more likely to convert to AD (Figure 4C). For whole-brain volume, those with volumes below the median value were more likely to convert to AD (Figure 4D).

## Discussion

We found that lower hippocampal and higher WMH volume predicted progression in controls who converted to MCI; lower hippocampal volume, CSF amyloid, whole-brain volume, and higher CSF ptau predicted progression from MCI to AD. Smaller hippocampi were a consistent predictor of clinical progression in both groups. Notably, models that included all variables were a better fit compared with separate models that individually investigated each marker of interest.

There was strong evidence that higher WMH volume and lower hippocampal volume were associated with conversion to MCI individually and independently. Lower whole-brain volume and lower CSF amyloid were associated with conversion to MCI when considered individually but not independently in our main analyses.

Past research has shown that CSF amyloid is an important predictor of cognitive impairment,^32^ and previous univariate models report a significant association with progression to MCI.^7^ This is consistent with our current findings in univariate models, and we extend this to show that this association is not significant when accounting for other biomarkers. This suggests that, in the cohort of individuals recruited as controls, other biomarkers (hippocampal volumes and WMH) may better identify those likely to progress to MCI.

We did not find evidence of ptau being predictive of future progression to MCI. There are mixed findings with respect to ptau, with some studies reporting a lack of evidence for ptau being a predictor in early disease stages and concluding that it may be useful later in the disease.^32^ It could be suggested that our CNtoMCI cohort may be too early in the disease to find ptau predictive. It is also possible that ptau was not a significant predictor in our models because individuals who convert to MCI in this group may not all be on the AD pathway. A substantial proportion of converters in this group may be following a more vascular or mixed pathology pathway, and this may explain the differences between our findings and previous work.^6,7^ As ptau in those with vascular dementia shows similar levels to healthy controls,^33^ this strengthens the suggestion that our CNtoMCI cohort is likely to contain a mixed population of those on AD, vascular, or other pathways.

The strong association of WMH with progression in this group may be consistent with these individuals being on a more vascular or mixed dementia pathway.^18,19^ Supportive evidence assessing patients with cerebral small vessel disease showed that WMH and hippocampal volume were useful in predicting dementia progression,^34^ congruent with our findings. Another study reported no association with WMH and progression.^20^ As they noted, we cannot be certain that those converting to MCI will progress to AD or another dementia type, nor can we be certain as to which clinical criteria they will meet if they do convert.

In the CSF model (eTable 1, links.lww.com/WNL/C477), CSF amyloid was significant. Without age as a covariate, microbleeds were individually associated with progression (eTable 3). The variability of significance of these 2 markers in the individual, fully adjusted, and supplementary analysis suggests that associations with other markers are likely present. For example, associations of CSF amyloid and WMH have been shown previously.^35^ As WMH and hippocampal volume are strong predictors, they may influence the association of CSF amyloid and microbleeds in larger models.

These results show higher WMH and lower hippocampal volume as strong predictors of clinical progression in healthy controls, both individually and in models that include other AD pathology biomarkers. Although we do not yet know whether these individuals will progress to AD in the future, using baseline WMH and hippocampal volume is useful in identifying those who develop cognitive impairment, and these findings may be useful in enriching clinical trials. Notably, models that included all variables were a better fit than individual models, suggesting biomarkers that capture a full range of pathology are more likely to identify individuals likely to progress.

Lower hippocampal volume, whole-brain volume, CSF amyloid, and higher CSF ptau were most strongly associated with conversion to AD in all models, including in supplementary models (eTable 2, links.lww.com/WNL/C477).

These results are consistent with hypothetical biomarker models,^3^ and previous univariate models of hippocampal volume,^36^ whole-brain volume,^12^ CSF ptau,^37^ and CSF amyloid,^38^ as predictors for conversion to AD. Confirmatory aspects of these results strengthen their individual use when identifying individuals likely to progress.

In the fully adjusted model without an age covariate, and when adjusting for age, sex, and education (eTables 5 and 6, links.lww.com/WNL/C477), whole-brain volume was not predictive of progression. This highlights the variability of baseline whole-brain volume as a clear predictor of progression to AD. Past reports of univariate models using baseline whole-brain volume have shown brain volume to be predictive,^12^ and other reports have shown no significant predictive power of this marker.^36,39^ Multivariable models that also include whole-brain atrophy do not report whole-brain volume as a significant predictor,^36,39^ as progressive atrophy is likely a stronger predictor. Discrepancies between our and previous work may be due to this marker's association with other covariates and a more subtle association with progression to AD. More specific regional markers, such as hippocampal volume, are more consistent predictors of clinical progression.

In multivariable models, there are differences between our and others' work. With CSF amyloid, previous research has reported no association in other multivariable models.^12^ As these previous models considered composite cognitive markers and did not consider cerebrovascular markers, this may suggest that cognitive markers, that are partly associated with amyloid deposition, may provide additional benefit in identifying those likely to progress. Future work could include these cognitive measures into our multivariable model to further explore these associations. An additional publication reporting a multivariable model also showed no association of hippocampal volume or CSF amyloid.^40^ As the study did not consider these markers individually, it is difficult to infer whether hippocampal volume and CSF amyloid were not predictive individually, or their associations were affected by other biomarkers included in their models.

Of interest, although the proportion of those with CMB and the WMH volume in those with AD has been reported to be higher than MCI,^41^ we did not find these markers to be associated with future progression. Moreover, the CSF and brain volume markers were independent of WMH and CMB in predicting progression to AD.

Our findings in MCItoAD confirm the hypothetical biomarker model and previous individual Cox regression models. It is important that we report the individual associations of key AD-related biomarkers (amyloid, tau, and neurodegeneration) with future diagnostic progression, their mutually independent predictive ability with each other, and presumed vascular markers. We also show that a model containing all variables is better at predicting conversion than the individual models and should be considered when identifying individuals with MCI likely to progress diagnostically in clinical trials.

ADNI excluded participants with significant CVD (determined by a Hachinski ischemic score of >4). Even with this limitation, we found that WMH were an important predictor of conversion of CN individuals to MCI. However, this may mean that we are underestimating the impact of CVD and its effects on progression. In MCItoAD, we may have missed a real effect of vascular disease in the current cohort that may be present in a more inclusive MCI group. It would be difficult to generalize these findings to a community-based population, specifically with the coexistence of vascular pathologies in both MCI and AD.

Lacunes could have been an important vascular marker to consider because of their association with neurodegeneration and WMH.^15,16^ ADNI excludes those with multiple lacunes and lacunes in critical memory structures. We found that the sample size with lacunes was too small to consider testing this variable formally.

The current cohort consists of mainly White and relatively well-educated individuals. This limits generalizability of our study to more diverse populations.

Most individuals in the current study with an MCI diagnosis have amnestic presentations. There is some debate around the use of the term “amnestic MCI” in ADNI,^42^ and future work could explore whether the level of memory impairment influences results within the ADNI cohort.

For multivariable Cox regression models, we chose to present data in complete cases. Excluding those with missing data may have biased our analysis in addition to reducing power to detect effects. This resulted in a smaller sample size and some instability that would benefit from future replication with a larger cohort.

Most importantly, we do not have autopsy confirmation of the diseases causing cognitive impairment. This remains the gold standard when confirming diagnosis.

With continued follow-up of the ADNI cohort, it may be possible to study individuals who progress from CN to MCI through to dementia. This would potentially allow us to make inferences regarding the earliest biomarkers of AD. It would also be important to consider those progressing to other vascular and mixed dementias, as we could then make accurate inferences regarding the significant predictors in the previously reported CNtoMCI and MCItoAD groups. By continuing to follow these individuals through their disease course, with more extensive diagnostic etiologies, vital information regarding disease progression, heterogeneity, and further biomarker predictions could be inferred.

Our work assessed baseline biomarkers of classical AD pathology, presumed CVD, and neurodegeneration. By fitting separate and mutually adjusted multivariable models, we were able to comprehensively assess associations of these biomarkers with clinical progression, both individually and independently of other biomarkers. Furthermore, this enabled us to establish that models including all variables were better at predicting progression than individual models. Our findings add to the existing literature on biomarkers that predict progression from control to MCI as well as from MCI to AD and will aid researchers when selecting the biomarkers needed to identify individuals likely to clinically progress.

The current study examined both univariate and multivariable Cox regression models of biomarkers that are likely useful in predicting clinical progression to AD. As previous studies have focused typically on univariate models, our novel research has expanded on this to demonstrate biomarkers that are independently predictive of conversion to MCI and AD, as well as demonstrate the associations the biomarkers have with each other in larger multivariate models.

This study showed that higher WMH and lower hippocampal volume predicted clinical conversion to MCI in those who were enrolled as controls, whereas higher ptau, lower hippocampal volume, and lower CSF amyloid predicted conversion to AD from MCI. Lower hippocampal volume is a consistent predictor of future clinical progression, which is likely due to it being a vulnerable structure to many pathologic insults, and reduction in its volume will affect memory and cognitive process associated with MCI and AD. Our results indicate that WMH are early biomarkers of future cognitive impairment in controls. This may be because the controls in this study who converted to MCI are on a mixed or non-AD pathologic pathway. Both in univariate and multivariate models, baseline WMH and hippocampal volume were meaningful predictors of conversion to MCI, emphasizing their importance to help identify those at risk of future clinical progression and identifying those for future trial participation.

## Glossary

AD: Alzheimer disease
ADNI: Alzheimer's Disease Neuroimaging Initiative
CDR: Clinical Dementia Rating
CMB: cerebral microbleeds
CN: cognitively normal
CVD: cerebrovascular disease
HR: hazard ratio
MCI: mild cognitive impairment
MMSE: Mini-Mental State Examination
SMC: significant memory concern
WMH: white matter hyperintensities
TIV: total intracranial volume

## References

[1] Dementia statistics [online]. alzint.org/about/dementia-facts-figures/dementia-statistics/. Accessed May 13, 2022.

[2] Jellinger KA. Clinicopathological analysis of dementia disorders in the elderly–an update. J Alzheimer's Dis. 2006;9(s3):61-70. doi: 10.3233/jad-2006-9s308 16914845

[3] Jack CR Jr, Knopman DS, Jagust WJ, et al. Tracking pathophysiological processes in Alzheimer's disease: an updated hypothetical model of dynamic biomarkers. Lancet Neurol. 2013;12(2):207-216. doi: 10.1016/s1474-4422(12)70291-0 23332364 PMC3622225

[4] Price JL, Morris JC. Tangles and plaques in nondemented aging and “preclinical” Alzheimer's disease. Ann Neurol. 1999;45(3):358-368. doi: 10.1002/1531-8249(199903)45:3<358::aid-ana12>3.0.co;2-x 10072051

[5] Seeburger JL, Holder DJ, Combrinck M, et al. Cerebrospinal fluid biomarkers distinguish postmortem-confirmed Alzheimer's disease from other dementias and healthy controls in the OPTIMA cohort. J Alzheimers Dis. 2015;44(2):525-539. doi: 10.3233/jad-141725 25391385

[6] Moghekar A, Li S, Lu Y, et al. CSF biomarker changes precede symptom onset of mild cognitive impairment. Neurology. 2013;81(20):1753-1758. doi: 10.1212/01.wnl.0000435558.98447.17 24132375 PMC3821715

[7] Roe CM, Fagan AM, Grant EA, et al. Cerebrospinal fluid biomarkers, education, brain volume, and future cognition. Arch Neurol. 2011;68(9):1145-1151. doi: 10.1001/archneurol.2011.192 21911695 PMC3203689

[8] Blennow K, Shaw LM, Stomrud E, et al. Predicting clinical decline and conversion to Alzheimer's disease or dementia using novel Elecsys Aβ (1-42), pTau and tTau CSF immunoassays. Sci Rep. 2019;9:19024. doi: 10.1038/s41598-019-54204-z 31836810 PMC6911086

[9] Manning EN, Leung KK, Nicholas JM, et al. A comparison of accelerated and non-accelerated MRI scans for brain volume and boundary shift integral measures of volume change: evidence from the ADNI dataset. Neuroinformatics. 2017;15(2):215-226. doi: 10.1007/s12021-017-9326-0 28316055 PMC5443885

[10] Tabatabaei-Jafari H, Shaw ME, Walsh E, Cherbuin N, AsDN Initiative. Regional brain atrophy predicts time to conversion to Alzheimer's disease, dependent on baseline volume. Neurobiol Aging. 2019;83:86-94. doi: 10.1016/j.neurobiolaging.2019.08.033 31585370

[11] Ridha BH, Barnes J, Bartlett JW, et al. Tracking atrophy progression in familial Alzheimer's disease: a serial MRI study. Lancet Neurol. 2006;5(10):828-834. doi: 10.1016/s1474-4422(06)70550-6 16987729

[12] Li S, Okonkwo O, Albert M, Wang MC. Variation in variables that predict progression from MCI to AD dementia over duration of follow-up. Am J Alzheimer's Dis. 2013;2(1):12-28. doi: 10.7726/ajad.2013.1002 PMC3919474 24524014

[13] Attems J, Jellinger KA. The overlap between vascular disease and Alzheimer's disease-lessons from pathology. BMC Med. 2014;12:206-212. doi: 10.1186/s12916-014-0206-2 25385447 PMC4226890

[14] Jellinger KA, Attems J. Challenges of multimorbidity of the aging brain: a critical update. J Neural Transm. 2015;122(4):505-521. doi: 10.1007/s00702-014-1288-x 25091618

[15] Wardlaw JM, Smith EE, Biessels GJ, et al. Neuroimaging standards for research into small vessel disease and its contribution to ageing and neurodegeneration. Lancet Neurol. 2013;12(8):822-838. doi: 10.1016/s1474-4422(13)70124-8 23867200 PMC3714437

[16] Ferreira D, Shams S, Cavallin L, et al. The contribution of small vessel disease to subtypes of Alzheimer's disease: a study on cerebrospinal fluid and imaging biomarkers. Neurobiol Aging. 2018;70:18-29. doi: 10.1016/j.neurobiolaging.2018.05.028 29935417

[17] Moscoso A, Rey-Bretal D, Silva-Rodríguez J, et al. White matter hyperintensities are associated with subthreshold amyloid accumulation. Neuroimage. 2020;218:116944. doi: 10.1016/j.neuroimage.2020.116944 32445880

[18] Smith EE, Egorova S, Blacker D, et al. Magnetic resonance imaging white matter hyperintensities and brain volume in the prediction of mild cognitive impairment and dementia. Arch Neurol. 2008;65(1):94-100. doi: 10.1001/archneurol.2007.23 18195145

[19] Silbert LC, Dodge HH, Perkins LG, et al. Trajectory of white matter hyperintensity burden preceding mild cognitive impairment. Neurology. 2012;79(8):741-747. doi: 10.1212/wnl.0b013e3182661f2b 22843262 PMC3421153

[20] Soldan A, Pettigrew C, Zhu Y, et al. White matter hyperintensities and CSF Alzheimer disease biomarkers in preclinical Alzheimer disease. Neurology. 2020;94(9):e950–e960. doi: 10.1212/wnl.0000000000008864 31888969 PMC7238945

[21] Sudre CH, Cardoso MJ, Bouvy WH, Biessels GJ, Barnes J, Ourselin S. Bayesian model selection for pathological neuroimaging data applied to white matter lesion segmentation. IEEE Trans Med Imaging. 2015;34(10):2079-2102. doi: 10.1109/tmi.2015.2419072 25850086

[22] Fiford CM, Sudre CH, Pemberton H, et al. Automated white matter hyperintensity segmentation using Bayesian model selection: assessment and correlations with cognitive change. Neuroinformatics. 2020;18(3):429-449. doi: 10.1007/s12021-019-09439-6 32062817 PMC7338814

[23] Gregoire SM, Chaudhary UJ, Brown MM, et al. The Microbleed Anatomical Rating Scale (MARS): reliability of a tool to map brain microbleeds. Neurology. 2009;73(21):1759-1766. doi: 10.1212/wnl.0b013e3181c34a7d 19933977

[24] NiftyMIDAS. Accessed November, 2022. http://cmic.cs.ucl.ac.uk/platform/niftk/current/html/index.html

[25] Leung KK, Barnes J, Modat M, et al. Brain MAPS: an automated, accurate and robust brain extraction technique using a template library. Neuroimage. 2011;55(3):1091-1108. doi: 10.1016/j.neuroimage.2010.12.067 21195780 PMC3554789

[26] Freeborough PA, Fox NC, Kitney RI. Interactive algorithms for the segmentation and quantitation of 3-D MRI brain scans. Comput Methods Programs Biomed. 1997;53(1):15-25. doi: 10.1016/s0169-2607(97)01803-8 9113464

[27] Jorge Cardoso M, Leung K, Modat M, et al. STEPS: Similarity and Truth Estimation for Propagated Segmentations and its application to hippocampal segmentation and brain parcelation. Med image Anal. 2013;17(6):671-684. doi: 10.1016/j.media.2013.02.006 23510558

[28] Cardoso MJ, Modat M, Wolz R, et al. Geodesic information flows: spatially-variant graphs and their application to segmentation and fusion. IEEE Trans Med Imaging. 2015;34(9):1976-1988. doi: 10.1109/tmi.2015.2418298 25879909

[29] Bertens D, Tijms BM, Scheltens P, Teunissen CE, Visser PJ. Unbiased estimates of cerebrospinal fluid β-amyloid 1-42 cutoffs in a large memory clinic population. Alzheimer's Res Ther. 2017;9:8. doi: 10.1186/s13195-016-0233-7 28193256 PMC5307885

[30] Bartlett JW, Frost C, Mattsson N, et al. Determining cut-points for Alzheimer's disease biomarkers: statistical issues, methods and challenges. Biomarkers Med. 2012;6(4):391-400. doi: 10.2217/bmm.12.49 22917141

[31] Schemper M, Wakounig S, Heinze G. The estimation of average hazard ratios by weighted Cox regression. Stat Med. 2009;28(19):2473-2489. doi: 10.1002/sim.3623 19472308

[32] Roe CM, Fagan AM, Grant EA, et al. Amyloid imaging and CSF biomarkers in predicting cognitive impairment up to 7.5 years later. Neurology. 2013;80(19):1784-1791. doi: 10.1212/wnl.0b013e3182918ca6 23576620 PMC3719431

[33] De Jong D, Jansen RWMM, Kremer BPH, Verbeek MM. Cerebrospinal fluid amyloid ss42/phosphorylated tau ratio discriminates between Alzheimer's disease and vascular dementia. J Gerontol Ser A Biol Sci Med Sci. 2006;61(7):755-758. doi: 10.1093/gerona/61.7.755 16870640

[34] Van Uden IW, Van Der Holst HM, Tuladhar AM, et al. White matter and hippocampal volume predict the risk of dementia in patients with cerebral small vessel disease: the RUN DMC study. J Alzheimers Dis. 2015;49(3):863-873. doi: 10.3233/jad-150573 26529206

[35] Walsh P, Sudre CH, Fiford CM, et al. CSF amyloid is a consistent predictor of white matter hyperintensities across the disease course from aging to Alzheimer's disease. Neurobiol Aging. 2020;91:5-14. doi: 10.1016/j.neurobiolaging.2020.03.008 32305782

[36] Henneman W, Sluimer JD, Barnes J, et al. Hippocampal atrophy rates in Alzheimer disease: added value over whole brain volume measures. Neurology. 2009;72(11):999-1007. doi: 10.1212/01.wnl.0000344568.09360.31 19289740 PMC2821835

[37] Ewers M, Buerger K, Teipel SJ, et al. Multicenter assessment of CSF-phosphorylated tau for the prediction of conversion of MCI. Neurology. 2007;69(24):2205-2212. doi: 10.1212/01.wnl.0000286944.22262.ff 18071141

[38] Sörensen A, Blazhenets G, Schiller F, Meyer PT, Frings L. Amyloid biomarkers as predictors of conversion from mild cognitive impairment to Alzheimer's dementia: a comparison of methods. Alzheimers Res Ther. 2020;12:155-156. doi: 10.1186/s13195-020-00721-3 33213489 PMC7678323

[39] Spulber G, Niskanen E, MacDonald S, et al. Whole brain atrophy rate predicts progression from MCI to Alzheimer's disease. Neurobiol Aging. 2010;31(9):1601-1605. doi: 10.1016/j.neurobiolaging.2008.08.018 18829136

[40] Nolze-Charron G, Mouiha A, Duchesne S, Bocti C; AsDN Initiative. White matter hyperintensities in mild cognitive impairment and lower risk of cognitive decline. J Alzheimers Dis. 2015;46(4):855-862. doi: 10.3233/jad-140618 26402625

[41] Fiford CM, Manning EN, Bartlett JW, et al. White matter hyperintensities are associated with disproportionate progressive hippocampal atrophy. Hippocampus. 2017;27(3):249-262. doi: 10.1002/hipo.22690 27933676 PMC5324634

[42] Duff K. Amnestic MCI in ADNI: maybe not enough memory impairment?. Neurology. 2021;97(12):595-596. doi: 10.1212/wnl.0000000000012587 34341152 PMC8480486

